# Salt intake reduction using umami substance-incorporated food: a secondary analysis of NHANES 2017–2018 data

**DOI:** 10.1017/S136898002200249X

**Published:** 2022-12-01

**Authors:** Shuhei Nomura, Shiori Tanaka, Akifumi Eguchi, Takayuki Kawashima, Haruyo Nakamura, Kaung Suu Lwin, Lisa Yamasaki, Daisuke Yoneoka, Yuta Tanoe, Megumi Adachi, Hitomi Hayabuchi, Shosei Koganemaru, Toshihide Nishimura, Byron Sigel, Hisayuki Uneyama, Kenji Shibuya

**Affiliations:** 1 Department of Global Health Policy, Graduate School of Medicine, The University of Tokyo, Tokyo, Japan; 2 Department of Health Policy and Management, School of Medicine, Keio University, 35 Shinanomachi, Shinjuku-ku, Tokyo 160-8582, Japan; 3 Tokyo Foundation for Policy Research, Tokyo, Japan; 4 Division of Prevention, National Cancer Center Institute for Cancer Control, Tokyo, Japan; 5 Centre for Preventive Medical Sciences, Chiba University, Chiba, Japan; 6 Department of Mathematical and Computing Science, Tokyo Institute of Technology, Tokyo, Japan; 7 School of Medicine, Nagasaki University, Nagasaki, Japan; 8 Infectious Disease Surveillance Center at the National Institute of Infectious Diseases, Tokyo, Japan; 9 Institute for Business and Finance, Waseda University, Tokyo, Japan; 10 Graduate School of Health and Environmental Sciences, Fukuoka Women’s University, Fukuoka, Japan; 11 Faculty of Nutrition, Kagawa Nutrition University, Saitama, Japan; 12 Division of Cancer Statistics Integration, Center for Cancer Control and Information Services, National Cancer Center, Tokyo, Japan; 13 Ajinomoto Co., Inc., Tokyo, Japan

**Keywords:** Sodium, Salt, Umami, USA

## Abstract

**Objective:**

Excessive salt intake raises blood pressure and increases the risk of non-communicable diseases (NCD), such as CVD, chronic kidney disease and stomach cancer. Reducing the Na content of food is an important public health measure to control the NCD. This study quantifies the amount of salt reduced by using umami substances, i.e. glutamate, inosinate and guanylate, for adults in the USA.

**Design:**

The secondary data analysis was performed using data of the US nationally representative cross-sectional dietary survey, the National Health and Nutrition Examination Survey (NHANES) 2017–2018. Per capita daily salt intake corresponding to the NHANES food groups was calculated in the four hypothetical scenarios of 0 %, 30 %, 60 % and 90 % market share of low-Na foods in the country. The salt reduction rates by using umami substances were estimated based on the previous study results.

**Setting:**

The USA

**Participants:**

4139 individuals aged 20 years and older in the USA

**Results:**

Replacing salt with umami substances could help the US adults reduce salt intake by 7·31–13·53 % (7·50–13·61 % for women and 7·18–13·53 % for men), which is equivalent to 0·61–1·13 g/d (0·54–0·98 g/d for women and 0·69–1·30 g/d for men) without compromising the taste. Approximately, 21·21–26·04 % of the US adults could keep their salt intake below 5 g/d, the WHO’s recommendation in the scenario where there is no low-Na product on the market.

**Conclusions:**

This study provides essential information that the use of umami substances as a substitute for salt may help reduce the US adults’ salt intake.

After smoking, the second major preventable behavioural risk factor for non-communicable disease (NCD) is unhealthy diet^(1)^. Among the unhealthy diet, excessive salt intake is one of the greatest contributors to the burden of NCD. High-salt diet raises blood pressure^(2)^, triggers CVD^(3,4)^ and chronic kidney disease^(5)^, and increases the risk of developing stomach cancer^(6,7)^. In 2019, approximately 1·9 million deaths worldwide were attributed to high-salt diet^(1)^, and the number of deaths attributed to the behavioural risk has increased by 42·8 % in the last 30 years^(8)^. Moreover, the reduction of salt intake is one of the nine targets in the NCD Global Monitoring Framework^(9)^ set by the WHO in 2013. Salt intake reduction is also known to be one of the most cost-effective or even cost-saving NCD control measures^(10)^. However, as of 2020, no country has achieved the goal of a 30 % reduction in salt intake between 2011 and 2025^(11)^.

High-salt diets are a major policy issue, especially in East Asian countries, Eastern European countries, and the USA^(8,12,13)^. While the WHO recommends daily salt intake of 5 g or less^(14)^, adults aged 20 years and older were consuming 8·97 g/d in the USA in 2017–2018^(15)^.

In recent years, the replacement of sodium chloride (NaCl, the chemical name for salt) with umami has been discussed as a healthy and natural solution to reduce salt intake^(16–18)^. Umami, which means pleasant savoury taste in Japanese, is induced by monosodium glutamate (MSG) and 5’-ribonucleotides, such as guanosine monophosphate and inosine monophosphate. The amount of Na in MSG, for example, is 12·28 g/100 g, that is 1/3 of that in NaCl (39·34 g/100 g)^(19)^. It is the fifth basic taste alongside the classical four basic tastes of saltiness, sweetness, bitterness and acidity^(20)^. However, few studies have been conducted to empirically evaluate the impact of umami on salt reduction at the population level. In this study, we examined the impact of incorporating umami into the daily salt intake of adults in the USA.

## Methods

### Study design and participants

We used anonymous secondary open data from the National Health and Nutrition Examination Survey (NHANES) for non-institutionalised adults aged 20 years and older between 2017 and 2018 in the USA. The NHANES, conducted by the National Center for Health Statistics (NCHS), is a cross-sectional survey with a stratified, multi-stage probability sample design. The NHANES collects 24-h dietary intake recalls for 2 d using the interview-administered Automated Multiple-Pass Method (AMPM) for a nationally representative sample over a 2-year study period^(21,22)^. The dietary intake could be either pre-packaged or prepared at home. The questionnaires, data sets and all related documents for each NHANES cycle are available on the NCHS website^(23)^.

### Demographic data

For the first day of interview, interviewers collected demographic information from the participants at each household, including their gender and ages. We created age groups as 20–29, 30–39, 40–49, 50–59, 60–69, 70–79 and 80+ years at 10-year intervals.

### Food and sodium intake data

All foods and beverages reported in the interviews were assigned a food code using the Food and Nutrient Database for Dietary Studies (FNDDS) 2017–2018 edition. The food code converts consumed foods and beverages reported in the interviews into gram quantities and determines the corresponding nutrient (e.g. Na) content. It should be noted that a previous study analysing 24-h urinary Na data collected using AMPM suggests that the method is a valid means of determining Na intake in adults^(24)^.

The FNDDS provides an eight-digit food code to uniquely identify each food/beverage. The first digit in the food code identifies one of nine major food groups: (1) milk, (2) meat and fish, (3) eggs, (4) legumes, nuts and seeds, (5) grains, (6) fruits, (7) vegetables, (8) fats, oils and salad dressings, and (9) sugars, sweets and beverages. The second and subsequent digits of the food code indicate the specific subgroups within the nine major food groups. In this study, all analyses were conducted at the subgroup level, but the estimation results are presented for the major food groups, separating fish and meat.

In this study, the average intake of each food group and the corresponding Na intake derived from the 2-d dietary interview was calculated and analysed as a daily value. Salt equivalent intake (g) was defined as Na (mg) × 2·54/1000. Please note that we did not apply the sampling weight in order to evaluate the distribution of daily salt intake on an individual basis to examine how much it changes before and after the incorporation of umami substances^(25)^.

### Sodium reduction rate in various food products with the incorporation of umami substances

According to scientific literature, the incorporation of umami substances can reduce Na in various food products, while maintaining their palatability. From inception to 6 April 2022, we searched for English-language articles that estimate the potential Na reduction rates by umami substances using the PubMed with the search terms (‘sodium intake’ OR ‘salt intake’ OR ‘sodium reduction’ OR ‘salt reduction’) AND (‘umami’ OR ‘MSG’ OR ‘monosodium glutamate’ OR ‘inosinate’ OR ‘CDG’ OR ‘calcium diglutamate’ OR ‘guanilate’ OR ‘guanylate’). The search strategy was iterative; however, we also explored bibliographies of potentially eligible studies to look for additional articles. Based on previous studies and input from several food and nutrition experts (co-authors), we estimated Na reduction rates for umami substances by NHANES food subgroups as listed in Table S1.

### Estimating salt intake reduction with the incorporation of umami substances

As people in the USA may already consume certain amounts of low-Na foods in their diet, we set four hypothetical scenarios in which 0 %, 30 %, 60 % and 90 % of food on the market is low-Na products. We assumed that the share of the low-Na food products on the market is same across all food groups, and that people consume the low-Na products at the same rate as these market shares. We calculated the possible amount of salt reduction at the population level for each major food group by the above-mentioned scenarios and gender. The Na reduction rate for each NHANES subgroup, expressed as an upper-lower interval in Table S1, represents the range of possible salt reduction rates estimated in the literature. The upper and lower limits were then used to calculate the maximum and minimum possible salt reduction for each subgroup at the individual level.

The following equations give the upper and lower limits of the *j*-th food subgroup-specific reduction in salt intake due to the incorporation of umami substances in the *i*-th individual.

Upper reduction in salt intake of the *j*-th item under the *k*th scenario in the *i*-th individual:

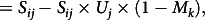




Lower reduction in salt intake of the *j*-th item under the *k*th scenario in the *i*-th individual:

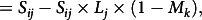

where 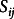 refers to the current salt intake of the *j*-th food subgroup in the *i*-th participant; 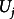 and 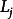 refer to the upper and lower limits of the salt reduction rate of the *j*-th food subgroup, and 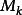 refers to the *k*-th scenario of the market share of low-Na products (denoted as 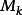 = 0, 0.3, 0.6 or 0.9 (*k* = 1, 2, 3, 4, respectively)).

Salt reduction was assumed to be zero in food groups when no evidence was found in the literature. After calculating the individual-level salt reduction for each scenario and subgroup using the above formula, we aggregated the amount of salt that could be reduced per major food group and calculated the average value at the population level.

We also calculated the percentage of the population that has already reached and would reach the WHO recommendation of daily salt intake (5 g/d) using umami substances in the four hypothetical scenarios by gender and age group^(26)^. R version 4.0.5 was used for all analyses.

## Results

The NHANES 2017–2018 cohort comprised a total of 5569 respondents aged 20 years and older (2867 women and 2702 men), with mean age of 51·50 and sd of 17·81. The analysis included a total of 4139 individuals (2162 women and 1977 men), with mean age of 51·36 and sd of 17·50, who had 2 d of dietary intake data on the usual amount of food consumed for both days. We excluded the respondents who did not complete the 2-d dietary intake data.

Table 1 shows the non-weighted gender- and age-group-specific mean daily salt intake, and the population percentage achieving the WHO recommendation salt intake level. These estimates exclude discretionary salt which was not recorded in NHANES. Women had a lower salt intake than men across all age groups. The mean daily salt intake was highest among women aged 20–29 years (8·03 g/d) and men aged 30–39 years (10·73 g/d), but lowest among those aged 80+ years for both women (6·40 g/d) and men (7·71 g/d). By age group, salt intake tended to be higher among younger than older persons. Of the total population, 17·18 % has already achieved the WHO recommendation.

**Table 1:** Demographic characteristics of the study participants and their current salt intake

Age (years)	Number of participants	Current mean salt intake* (g/d)		Percentage of people below WHO‡ recommendation (%)
Total population		Mean	sd †	
20–29	585	9·14	4·17	13·68
30–39	647	9·04	4·19	12·52
40–49	615	8·87	3·92	15·28
50–59	706	8·24	3·73	17·14
60–69	869	7·89	3·49	19·91
70–79	463	7·46	3·34	23·97
80+	254	7·03	2·46	20·08
All	4139	8·35	3·80	17·18
Women				
20–29	304	8·03	3·40	18·75
30–39	362	7·70	3·14	17·40
40–49	329	7·42	3·33	23·71
50–59	381	7·04	2·78	23·10
60–69	431	6·76	2·77	28·07
70–79	223	6·51	2·82	33·18
80+	132	6·40	2·19	27·27
All	2162	7·20	3·04	23·91
Men				
20–29	281	10·34	4·58	8·19
30–39	285	10·73	4·72	6·32
40–49	286	10·54	3·89	5·59
50–59	325	9·63	4·19	10·15
60–69	438	9·01	3·75	11·87
70–79	240	8·35	3·54	15·42
80+	122	7·71	2·55	12·30
All	1977	9·61	4·14	9·81

* Current mean salt intake is non-weighted. These estimates exclude discretionary salt which was not recorded in NHANES.

† Standard deviation.

‡ World Health Organisation.

The amount of salt intake that is possibly reduced by using umami substances for NHANES major food groups is presented under the four scenarios in Table 2. For the scenario that assumes no low-Na products on the market, the highest amount of expected salt reduction was identified in vegetable (0·24–0·35 g/d), followed by milk (0·18–0·33 g/d) and meat (0·11–0·42 g/d). The total amount of salt reduction across all major food groups in the scenarios, that 0 %, 30 %, 60 % and 90 % of food on the market is low-Na products, was 0·61–1·13 g/d, 0·43–0·79 g/d, 0·24–0·45 g/d and 0·06–0·11 g/d, respectively.

**Table 2:** Estimated lower-upper mean reduction in salt intake using umami substances by market share scenarios of low-sodium products, gender and the NHANES* major food groups

			Estimated lower-upper mean reduction in salt intake (g/d)			
	Current mean salt intake (sd †) (g/d)		Scenario 1 (current market share of low-Na products = 0 %)	Scenario 2 (current market share of low-Na products = 30 %)	Scenario 3 (current market share of low-Na products = 60 %)	Scenario 4 (current market share of low-Na products = 90 %)
Milk	Mean	sd †				
Total population	0·56	0·61	0·18–0·33	0·13–0·23	0·07–0·13	0·02–0·03
Women	0·52	0·56	0·17–0·31	0·12–0·22	0·07–0·12	0·02–0·03
Men	0·61	0·67	0·19–0·35	0·13–0·24	0·08–0·14	0·02–0·03
Meat						
Total population	2·61	1·96	0·11–0·42	0·08–0·30	0·05–0·17	0·01–0·04
Women	2·08	1·50	0·09–0·33	0·06–0·23	0·04–0·13	0·01–0·03
Men	3·16	2·22	0·14–0·52	0·10–0·37	0·06–0·21	0·01–0·0
Fish						
Total population	1·14	1·13	0·01–0·01	0·00–0·00	0·00–0·00	0·00–0·00
Women	1·02	1·03	0·00–0·00	0·00–0·00	0·00–0·00	0·00–0·00
Men	1·31	1·23	0·01–0·01	0·01–0·01	0·00–0·00	0·00–0·00
Eggs						
Total population	0·54	0·52	NA‡	NA	NA	NA
Women	0·46	0·44	NA	NA	NA	NA
Men	0·62	0·58	NA	NA	NA	NA
Legumes, nuts and seeds						
Total population	0·70	0·88	0·03–0·04	0·02–0·03	0·01–0·02	0·00–0·00
Women	0·60	0·81	0·02–0·04	0·02–0·03	0·01–0·02	0·00–0·00
Men	0·82	0·95	0·03–0·04	0·02–0·03	0·01–0·02	0·00–0·00
Grains						
Total population	3·06	2·28	0·10	0·07	0·04	0·01
Women	2·69	1·90	0·09	0·06	0·04	0·01
Men	3·47	2·59	0·10	0·07	0·04	0·01
Fruits						
Total population	0·04	0·13	NA	NA	NA	NA
Women	0·04	0·15	NA	NA	NA	NA
Men	0·03	0·10	NA	NA	NA	NA
Vegetables						
Total population	1·08	1·08	0·24–0·35	0·17–0·24	0·10–0·14	0·02–0·03
Women	0·97	0·91	0·20–0·30	0·14–0·21	0·08–0·12	0·02–0·03
Men	1·19	1·23	0·28–0·40	0·19–0·28	0·11–0·16	0·03–0·04
Fats, oils and salad dressings						
Total population	0·39	0·50	0·03	0·02	0·01	0
Women	0·36	0·46	0·03	0·02	0·01	0
Men	0·41	0·54	0·03	0·02	0·01	0
Sugar, sweets and beverages						
Total population	0·31	0·35	NA	NA	NA	NA
Women	0·27	0·29	NA	NA	NA	NA
Men	0·36	0·41	NA	NA	NA	NA
All foods						
Total population	8·35	3·80	0·61–1·13	0·43–0·79	0·24–0·45	0·06–0·11
Women	7·20	3·04	0·54–0·98	0·38–0·68	0·22–0·39	0·05–0·10
Men	9·61	4·14	0·69–1·30	0·48–0·91	0·27–0·52	0·07–0·13

* NHANES, National Health and Nutrition Examination Survey.

† Standard deviation.

‡ NA referrers to no evidence on the salt reduction with the incorporation of umami substances.

Table 3 presents the estimated salt intake when umami substances were used as substitute for salt by gender and age group under the four scenarios. The mean daily salt intake when umami substances were used was estimated to be 7·22–7·74 g/d, 7·56–7·92 g/d, 7·90–8·11 g/d and 8·24–8·29 g/d under the scenarios that 0 %, 30 %, 60 % and 90 % of food on the market was low-Na products, respectively. Additionally, the percentages of women and men who would achieve the WHO recommendation using umami substances in the scenarios that 0 %, 30 %, 60 % and 90 % of food on the market is low-Na products were 29·42–35·11 % and 12·24–16·14 %; 28·08–31·22 % and 11·43–13·40 %; 26·09–27·89 % and 10·67–11·73 %; and 24·24–24·70 % and 9·91–9·96 %, respectively.

**Table 3:** Estimated lower-upper interval of salt intake and the percentage that the US* population would reach the WHO recommendation of daily salt intake (5 g/d) when umami substances are used as substitute for salt in the four hypothetical scenarios by gender and age group

	Estimated lower-upper interval of mean salt intake (g/d), percentage of people below WHO† recommendation (%)							
Age (years)	Scenario 1	%	Scenario 2	%	Scenario 3	%	Scenario 4	%
Total population								
20–29	8·05–8·52	16·07–20·00	8·38–8·71	15·38–16·75	8·71–8·89	15·04–15·73	9·03–9·08	13·68–13·85
30–39	7·88–8·41	16·54–20·09	8·23–8·60	15·46–17·93	8·58–8·79	13·91–14·84	8·92–8·98	12·83–12·98
40–49	7·72–8·23	18·37–22·60	8·07–8·42	17·72–19·67	8·41–8·61	16·42–18·21	8·75–8·81	15·77–15·93
50–59	7·11–7·62	22·38–26·91	7·45–7·81	20·82–23·80	7·78–7·99	18·41–20·40	8·12–8·17	17·28–17·56
60–69	6·74–7·28	23·36–29·11	7·09–7·47	22·55–24·97	7·43–7·65	21·86–22·78	7·78–7·83	20·02–20·48
70–79	6·27–6·85	30·02–35·21	6·63–7·03	28·29–31·97	6·98–7·22	25·92–28·51	7·34–7·40	24·41–24·41
80+	6·06–6·51	25·20–33·86	6·35–6·67	23·62–28·35	6·64–6·82	22·05–24·02	6·93–6·98	20·08–20·87
All	7·22–7·74	21·21–26·04	7·56–7·92	20·13–22·71	7·90–8·11	18·72–20·71	8·24–8·29	17·40–17·66
Women								
20–29	7·05–7·46	21·05–25·33	7·34–7·63	20·72–21·71	7·64–7·81	20·72–21·05	7·93–7·98	18·75–19·08
30–39	6·70–7·14	23·48–27·62	7·00–7·31	21·82–24·86	7·30–7·48	19·34–20·72	7·60–7·65	17·68–17·96
40–49	6·42–6·86	28·57–33·13	6·72–7·03	27·36–30·09	7·02–7·20	25·23–28·27	7·32–7·36	24·32–24·62
50–59	6·06–6·49	30·71–36·48	6·35–6·66	28·35–32·28	6·65–6·82	24·93–27·56	6·95–6·99	23·36–23·88
60–69	5·80–6·24	32·48–39·21	6·09–6·39	32·02–34·57	6·38–6·55	30·86–31·79	6·66–6·71	28·31–29·00
70–79	5·55–5·99	40·81–47·09	5·84–6·15	39·01–43·05	6·13–6·30	36·32–38·57	6·41–6·46	34·08–34·08
80+	5·47–5·89	34·09–45·45	5·75–6·05	31·82–39·39	6·13–6·30	29·55–32·58	6·31–6·35	27·27–28·79
All	6·22–6·65	29·42–35·11	6·51–6·82	28·08–31·22	6·81–6·98	26·09–27·89	7·10–7·15	24·24–24·70
Men								
20–29	9·14–9·66	10·68–14·23	9·50–9·86	9·61–11·39	9·86–10·07	8·90–9·96	10·22–10·27	8·19–8·19
30–39	9·38–10·02	7·72–10·53	9·78–10·24	7·37–9·12	10·19–10·45	7·02–7·37	10·60–10·66	6·67–6·67
40–49	9·22–9·81	6·64–10·49	9·61–10·03	6·64–7·69	10·01–10·24	6·29–6·64	10·40–10·46	5·94–5·94
50–59	8·34–8·95	12·62–15·69	8·73–9·15	12·00–13·85	9·11–9·36	10·77–12·00	9·50–9·56	10·15–10·15
60–69	7·67–8·32	14·38–19·18	8·07–8·52	13·24–15·53	8·47–8·73	13·01–13·93	8·87–8·94	12·10–11·87
70–79	6·93–7·65	20·00–24·17	7·36–7·86	18·33–21·67	7·78–8·07	16·25–19·17	8·21–8·28	15·42–15·42
80+	6·70–7·18	15·57–21·31	7·00–7·18	14·75–16·39	7·31–7·50	13·93–14·75	7·61–7·66	12·30–12·30
All	8·31–8·92	12·24–16·14	8·70–9·13	11·43–13·40	9·09–9·33	10·67–11·73	9·48–9·54	9·91–9·96

* United States.

† World Health Organisation.

## Discussion

The incorporation of umami substances into certain foods could potentially reduce daily salt intake by 7·31–13·53 %, which is equivalent to 0·61–1·13 g/d at the population level in the USA. A previous study that used the NHANES 2013–2016 data showed that salt intake could be reduced by 7·3 % by replacing salt with MSG^(27)^. In comparison, our study expanded the scope of study from MSG to umami substances, which includes MSG and 5’-ribonucleotides, such as guanosine monophosphate and inosine monophosphate, and selected the wider range of foods. As such, our findings suggested umami substances have a greater potential to reduce salt intake than in the previous study. Also, our study found that the replacement of salt in meat products has the greatest impact on reducing daily salt intake by up to 0·11–0·42 g/d (4·21–16·09 %).

On the other hand, global recognition of MSG as an effective and practical solution for salt reduction remains a major challenge. The study in 1968 reported that MSG in Chinese food has caused numbness and palpitations in the neck and arms, and it is linked to various health problems, known as the Chinese restaurant syndrome^(28)^. Following this study, several studies also reported the association between MSG and various health effects, including asthma, urticaria, atopic dermatitis, dyspnea, tachycardia, metabolic syndrome, obesity and blood pressure increase^(29–33)^. However, other studies, including a double-blind placebo-controlled trial, have evaluated the reported reactions to MSG and confirmed a lack of plausible evidence between MSG intake and the development of such symptoms^(34–37)^. Furthermore, major scientific committees and regulatory bodies, such as the US Food and Drug Administration (FDA), the Joint FAO/WHO Expert Committee on Food Additives (JECFA) and the European Commission Scientific Committee on Food (SCF), have assessed the safety of MSG, and all separately came to a conclusion that MSG is safe to consume at a normal intake level and there is no evidence linking the use of MSG to long-term medical problems for the general public^(38)^.

Public measures, such as a nutrition labelling system alone, may not sufficiently reduce daily salt intake because lowering Na intake may not be a priority among consumers^(39)^. It might be difficult to reduce Na content in food if it affects their palatability^(40)^. Therefore, food industries should make efforts to adapt low-Na food products to the consumer preferences^(41)^. In this context, combining umami substances with other flavours of food might be an effective way to reduce salt intake^(42,43)^. Umami substances enhance the flavour of food itself, and consumers should accept umami substances since they are naturally present in various foods^(44)^.

Our study has several strengths and limitations. The strength of our analysis is that we used the data of the NHANES, a large, nationally representative sample, which allowed us to estimate average salt intake at the population level. In addition to the previously described limitations with NHANES data^(45–47)^, this study has the following limitations. First, this study used existing literature to determine salt reduction rates by using umami substances. The evidence may not be sufficient for all food groups. Second, we assumed the same share of the low-Na food products on the market across all food groups, though it is not likely the case in real settings. Third, the intake and usage of low-Na products may differ depending on the place where the meal is served or prepared (e.g. home or restaurant). However, this was not considered in this study due to insufficient data that allow us to properly examine these factors. Finally, we were unable to consider the acceptability of umami substances among consumers in the US to fully demonstrate the effects of salt reduction^(18,48,49)^.

## Supplementary material

For supplementary material accompanying this paper visit https://doi.org/10.1017/S136898002200249X. click here to view supplementary material
