# Supplementary material for: Salt intake reduction using umami substance-incorporated food: a secondary analysis of NHANES 2017–2018 data
Source: Public Health Nutr. 2022 Dec 1;26(2):488–95. doi: 10.1017/S136898002200249X (PMC13076077; doi:10.1017/S136898002200249X)
Supplement: Supplementary file 1 [file S136898002200249Xsup001.docx]

**Table S1.** Sodium reduction assumptions by using umami substances for the FNDDS^1^ subgroups

| Main group | Subgroup | FNDDS food code | Sodium reduction rate (%) | References | Umami substance |
| --- | --- | --- | --- | --- | --- |
| Milk | Cheese | 14XXXXXX^4^ | 54–100% | Rodriques (2014)^(1)^, da Silva (2014)^(2)^ | MSG^2^ |
|  | Savoury yoghurt dip | 114XXXXX | 46% | Halim (2020)^(3)^ | MSG |
|  | Requeijão cheese | 143XXXXX | 25-100% | Reis Rocha RA (2021)^(4)^ | MAG, MSG, GMP, IMP |
| Meat | Sausage | 252XXXXX | 17–75% | Wooward (2003)^(5)^, Ichikawa Chemical Institution (1984)^(6)^, dos Santos (2014)^(7)^, Campangol (2017)^(8)^ | MSG, CDG^3^, Inosinate, Trans-glutaminase, and guanylate |
|  | Chicken broth | 283XXXXX, 285XXXXX | 11–38% | Chi (1992)^(9)^, Carter (2011)^(10)^, Wang (2019)^(11)^ | MSG, CDG |
|  | Pork cauliflower fried rice | 2732XXXX | 61% | Halim (2020)^(3)^ | MSG |
|  | Beef burger | 2731XXXX | 25-100% | Reis Rocha RA (2021)^(4)^ | MAG, MSG, GMP, IMP |
|  | Dry-cured rabbit legs | 200XXXXX | 46% | Pedro D (2021)^(12)^ | MSG |
|  | Chicken rice | 2724XXXX | 40% | Leong (2016)^(13)^ | MSG |
|  | mee soto broth  Spicy soups (curry chicken and chilli chicken) | 2834XXXX | 33-40% | Jinap (2016)^(14)^  Leong (2016)^(13)^ | MSG |
| Fish | Salted fish | 26109170, 26109180 | 30–40% | Ichikawa Chemical Institution (1984) ^(6)^ | MSG, Inosinate |
|  | Fish burger | 2735XXXX | 50% | de Quadros (2015)^(15)^ | MSG |
| Legume | Miso | 41420110 | 15–35% | Ishida (2011)^(16)^, Yamasa Corporation (2014)^(17)^ | MSG, Inosinate, Guanilate |
|  | Soy sause | 41420300 | 40–61% | Kameda Seika Co., Ltd. (1997)^(18)^ | MSG, Inosinate, Guanilate |
| Grain | Snack | 540XXXXX, 543XXXXX, 544XXXXX | 51% | Buechler (2019)^(19)^ | MSG, Inosinate, Guanilate |
| Vegetable | Vegetable soup | 718XXXXX, 723XXXXX, 735XXXXX, 746XXXXX, 756XXXXX, 775XXXXX | 17–40% | Kremer (2009)^(20)^, Ball (2002)^(21)^, Hartley (2020)^(22)^ | Glutamates, CDG |
|  | Potate chips | 712XXXXX | 30% | Kongstad (2020)^(23)^ | MSG |
|  | Salted vegetable | 755XXXXX | 55% | Tampei Pharmaceutical Co., Ltd. (1985)^(24)^ | MSG |
|  | Roasted vegetable | 752XXXXX, 753XXXXX, 754XXXXX | 31% | Halim (2020)^(3)^ | MSG |
|  | Quinoa bowl | 77XXXXXX | 31% | Halim (2020)^(3)^ | MSG |
|  | Shoestring potatoes | 714XXXXX | 25-75% | Reis Rocha RA (2021)^(4)^ | MAG, MSG, GMP, IMP |
|  | Garlic and salt spice | 755XXXXX | 25-50% | Rodrigues (2014)^(25)^ | MSG |
|  | Tomato sauce | 744XXXXX | 50% | Rogerio (2020)^(26)^ | MSG, Inosinate, Guanilate |
|  | A traditional Japanese clear soup (sumashi-jiru) | 41601070 | 40% | Yamaguchi and Takahashi (1984)^(27)^ | MSG |
| Oil | Butter | 81101XXX | 100% | de Souza (2014)^(28)^ | MSG |
|  | Margarine | 81102XXX | 47% | Gonçalves (2017)^(29)^ | MSG |

Notes: 1 FNDDS: Food and Nutrient Database for Dietary Studies; 2 MSG: monosodium glutamate; 3 CDG: calcium diglutamate; 4 X refers to all food codes for which the first one or two digits correspond.

**References**

1. Rodrigues JF, Goncalves CS, Pereira RC *et al*. (2014) Utilization of temporal dominance of sensations and time intensity methodology for development of low-sodium Mozzarella cheese using a mixture of salts. J Dairy Sci **97**, 4733–44.
2. da Silva TLT, de Souza VR, Pinheiro ACM *et al*. (2014) Equivalence salting and temporal dominance of sensations analysis for different sodium chloride substitutes in cream cheese. Int J Dairy Technol **67**, 31–8.
3. Halim J, Bouzari A, Felder D *et al*. (2020) The Salt Flip: Sensory mitigation of salt (and sodium) reduction with monosodium glutamate (MSG) in "Better-for-You" foods. J Food Sci **85**, 2902–14.
4. Reis Rocha RA, Reis Rocha LC, Ribeiro MN *et al*. (2021) Effect of the food matrix on the capacity of flavor enhancers in intensifying salty taste. J Food Sci **86**, 1022–32.
5. Woodward DR, Lewis PA, Ball PJ *et al*. (2003) Calcium glutamate enhances acceptability of reduced-salt sausages. Asia Pac J Clin Nutr **12**, S35.
6. Ichikawa Chemical Institution. (1984) Low-salted processed meat. Japan Patent JPA59–118038.
7. dos Santos BA, Campagnol PCB, Morgano MA *et al*. (2014) Monosodium glutamate, disodium inosinate, disodium guanylate, lysine and taurine improve the sensory quality of fermented cooked sausages with 50% and 75% replacement of NaCl with KCl. *Meat Science* **96**, 509–13.
8. Campagnol P, Dos Santos BA, Lorenzo JM *et al*. (2017) A combined approach to decrease the technological and sensory defects caused by fat and sodium reduction in Bologna-type sausages. Food Sci Technol Int **23**, 471–9.
9. Chi SP, Chen TC. (1992) Predicting optimum monosodium glutamate and sodium chloride concentrations in chicken broth as affected by spice addition. J Food Process. Preserv **16**, 313–26.
10. Carter BE, Monsivais P, Drewnowski A. (2011) The sensory optimum of chicken broths supplemented with calcium di-glutamate: A possibility for reducing sodium while maintaining taste. Food Qual Prefer **22**, 699–703.
11. Wang S, Tonnis BD, Wang ML *et al*. (2019) Investigation of monosodium glutamate alternatives for content of Umami substances and their enhancement effects in chicken soup compared to monosodium glutamate. J Food Sci **84**, 3275–83.
12. Pedro D, Saldaña E, Lorenzo JM *et al*. (2021) Low-sodium dry-cured rabbit leg: A novel meat product with healthier properties. *Meat Science* **173**, 108372.
13. Leong J, Kasamatsu C, Ong E et al. (2015) A study on sensory properties of sodium reduction and replacement in Asian food using difference-from - control test. Food Sci Nutr **4**, 469–78.
14. Jinap S, Hajeb P, Karim R *et al*. (2016) Reduction of sodium content in spicy soups using monosodium glutamate. Food Nutr Res **60**, 30463.
15. De Quadros DA, de Oliveira Rocha IF, Ferreira SMR *et al*. (2015) Low-sodium fish burgers: Sensory profile and drivers of liking. LWT Food Sci Technol **63**, 236–42.
16. Ishida M, Tezuka H, Hasegawa T *et al*. (2011) Sensory evaluation of a low-salt menu created with umami, similar to savory, substance. Nippon Eiyo Shokuryo Gakkaishi **64**, 305–11.
17. Yamasa Corporation. (2014) Low-salt bean miso with excellent taste. Japan Patent JPA5523618.
18. Kameda Seika Co., Ltd. (1997) Low-salt, low-protein, low-phosphorus, low-potassium soy sauce-like seasoning. Japan Patent JPA09–275930.
19. Buechler AE, Lee SY. (2019) Consumer acceptance of reduced sodium potato chips and puffed rice: how does ingredient information and education influence liking? J Food Sci **84**, 3763–73.
20. Kremer S, Mojet J, Shimojo R. (2009) Salt Reduction in Foods Using Naturally Brewed Soy Sauce. J Food Sci **74**, S255–S62.
21. Ball P, Woodward D, Beard T *et al*. (2002) Calcium diglutamate improves taste characteristics of lower-salt soup. Eur J Clin Nutr **56**, 519–23.
22. Hartley IE, Liem DG, Keast RS. (2020) Females' ability to discriminate MSG from NaCl influences perceived intensity but not liking of MSG added vegetable broths. J Food Sci **85**, 3934–42.
23. Kongstad S, Giacalone D. (2020) Consumer perception of salt-reduced potato chips: Sensory strategies, effect of labeling and individual health orientation. Food Qual Prefer **81**, 103856.
24. Tampei Pharmaceutical Co., Ltd. (1985) Low sodium instant pickles. Japan Patent JPA60–153751.
25. Rodrigues JF, Junqueira G, Gonçalves CS *et al*. (2014) Elaboration of garlic and salt spice with reduced sodium intake. An Acad Bras Cienc **86**, 2065–75.
26. Rogério Tavares Filho E, Almeida Esmerino E, de Almeida Santos-Junior V *et al*. (2020) Dynamic aspects of salt reduction in tomato sauce by use of flavor enhancers and a bitter blocker. Food Sci Technol Int **26**, 549–59.
27. Yamaguchi S, Takahashi C. (1984) Interactions of monosodium glutamate and sodium chloride on saltiness and palatability of a clear soup. J Food Sci **49**, 82–5.
28. de Souza VR, Freire TVM, Saraiva CG et al. (2013) Salt equivalence and temporal dominance of sensations of different sodium chloride substitutes in butter. J Dairy Res **80**, 319–25.
29. Gonçalves C, Rodrigues J, Júnior H *et al*. (2017) Sodium reduction in margarine using NaCl substitutes. An Acad Bras Ciênc **89**, 2505–13.
